# Editors’ Book Table

**Published:** 1874-06

**Authors:** 


### Editors' Book Table.

NOTE.—All works reviewed in the columns of the CHICAGO MEDICAL JOURNAL may be found in the extensive stock of W. B. KEEN, COOKE & Co., whose catalogue of Medical Books will be sent to any address upon request.

*A Practical Treatise on the Diseases of Children.* By J. FORSYTH MEIGS, M.D., and WILLIAM PEPPER, M.D. Fifth edition, revised and enlarged. Philadelphia: Lindsay & Blakiston. 1874.

Our limits permit us but a brief notice of this admirable work. It has been practically criticised by the profession at large long and thoroughly, the verdict being a demand for a fifth edition.

A careful examination of the book will satisfy the honest reviewer that it is the result of the most laborious and extensive investigation, affording a comprehensive summary of the state of medical science of to-day. The most captious critic will weary in his search for defects in this most valuable contribution to American medical literature.

*Annual Report of the Supervising Surgeon of the Marine Hospital Service of the United States, for the fiscal year* 1873. By JNO. M. WOODWORTH, M.D. Washington: Government Printing Office. 1873.

The above report is a fair exhibit of what may be accomplished by industry, honesty, fearlessness, and good administrative ability, in the service of the General Government, and suggests the immense gain which would accrue from the application of the same qualities in all other departments of the Government, in many of which they have been sadly wanting.

The advantages resulting are best summed up in the language of the Report, viz:

To *increase* the number of customs districts in which hospital relief is furnished, nearly 27 per cent.

To *reduce* the average daily *per capita* cost of such relief about 20 per cent.

To *increase* the collection of hospital dues 16 per cent.

To *reduce* the total cost of the service 9.4 per cent.

To *reduce* the net cost to the Government over 51 per cent.; and, as a consequence,

To *diminish* the amount of the deficiency appropriation for the current year to only 40 per cent. of that of 1871.

In the chapter on "Hospitals and Hospital Construction," Dr. Woodworth, we are glad to say, condemns the old system of construction, upon hygienic principles, and compares the recently completed illustration of feudalism and the dark ages, called the Marine Hospital, at Chicago, with the cheap, airy and healthy Pavilion Hospital at San Francisco, very much to the disadvantage of the former, which cost enough to build eight of the latter.

Dr. Woodworth cannot confer a greater boon to his old home, Chicago, than by impressing his views upon this subject upon the County Commissioners of Cook County, pending their protracted deliberations upon the best mode of constructing the County Hospital.

The Report is supplemented by a map, accompanied by a description of the distribution and natural history of yellow fever, as it has occurred at different times in the United States, by J. M. Toner, M.D., President of the American Medical Association, Washington, D. C.; the map indicating not only the localities, but also the elevation at which the disease prevailed above the sea level.

Appended to the Report are the details, with photographic illustrations, of certain cases of injuries, so that the Marine Hospital Department, for the first time, is made, under Dr. Woodworth's able administration, to contribute something of its quota to the advancement of medical knowledge. H.

*The Puerperal Diseases.* Clinical Lectures delivered at Bellevue Hospital, by FORDYCE BARKER, M.D., Clinical Professor of Midwifery and the Diseases of Women, in the Bellevue Hospital and Medical College, etc. New York: D. Appleton & Co. 1874.

Few men in the country are better fitted, by innate ability, general accomplishment, and practical experience, to write authoritatively upon the subject of puerperal diseases than the author of this treatise; and it is with no small pride that we recognize our distinguished countryman as the pioneer in this department of English medical literature.

The work derives additional value in being not so much a transcript of the status of scientific opinion upon the important subject which it embraces, as a digest of clinical experience, the record of observation at the bedside, and the inductions therefrom.

The work is comprised in twenty-two lectures, in which all, or nearly all, the incidents and accidents of the puerperal state are elaborately discussed.

The subject of puerperal convulsions receives, as it merits, special attention, and we are glad to see that the author rejects the theory (of Braun) of the essential relations of albuminuria, uræmia and convulsions; for, while their frequent coincidence is undoubted, it is likewise true that either of these conditions may occur without the other. Moreover, the author takes another step in advance in the pathology of this terrible malady, by indicating the nervous rather than the vascular system as the starting point of the convulsive phenomena. It is to be regretted that he has not defined more clearly the pathological relations between the convulsion and the cerebral congestion which accompanies its subsidence, and which is by too many still regarded as an essential factor in the production of the convulsion.

Now, the two conditions, cerebral hyperæmia and convulsion, are essentially antagonistic, as has been demonstrated repeatedly by experiment; and, while the hyperæmia in this case constitutes a phase in the pathology of the disease of vital importance, in view of its consequences, it is, nevertheless, but an epiphenomenon of the convulsion, and has a distinctly secondary relation thereto. The establishment of the true pathological relations of the various phases of this fearful disease will alone clear the way to a rational therapeusis.

While there are, undoubtedly, differences in degree and intensity, we are totally unable to perceive any essential difference between puerperal convulsion and epilepsy; the puerperal state, in the one case, acting as does the neurotic diathesis in the other, by inducing a hyperæsthetic condition of the excitable ganglia of the cerebro-spinal system, awaiting only the application of an exciting cause to arouse its fullest morbid activity.

The chapters upon Puerperal Mania are very satisfactory. The author is very decided in his recognition of moral and mental influences as the most efficient agents in the production of this condition. He is likewise very decided in his opposition to all depletory or debilitating remedies; and, recognizing clearly debility as a constant factor in these conditions, teaches the necessity of restorative measures.

Dr. Barker rejects the theory of the traumatic or septicæmic origin of puerperal fever, which he considers as idiopathic, and to which the local inflammatory lesions bear analogous relations to those sustained by the pustules of small pox (for example) to the fever of that disease.

The line of demarcation between puerperal metritis and peritonitis and puerperal fever is very strongly drawn by the author—stronger, perhaps, than the evidence furnished by the authorities, so abundantly reported, would seem to justify.

His "confession of faith" upon this subject is admirably formulated into eight distinct propositions, which form an appropriate conclusion to the work of an able physician and an accomplished scholar, and which is destined to take its place among the classics of the profession.

The weary-eyed student, when he closes the book, will thank the Messrs. Appleton & Co. for the typographical perfection, which must commend it to all who appreciate the æsthetics of the book-maker's art. H.

*A Clinical History of the Medical and Surgical Diseases of Women.* By ROBERT BARNES, M.D., London, Fellow and Lumleian Lecturer (1873), Royal College of Physicians, etc. With one hundred and sixty-nine illustrations. Philadelphia: Henry C. Lea. 1874.

The field of gynæcology has become so comprehensive that a mere cursory glance at its more prominent landmarks, and a superficial description of its outlines, are all that are admissible in a journal of such restricted limits as our own. The magnitude of the subject has been firmly impressed upon our consciousness by an examination of the work of the great English gynæcologist.

The first five chapters are occupied with descriptions of the sexual organs, in which the points most noteworthy are the reiteration by the author of the opinions of Raney, Velpeau and Maygrier, of the use of the round ligaments as adjuncts in copulation, drawing, by their combined action, the uterus nearer to the symphysis pubis, thereby lengthening the vagina by means of the voluntary muscular fibres with which they are provided; and, further, the rejection of Robert Lee's demonstration of the uterine nerves, as contradicted by the later investigations of Hirschfeld, Richet, Boulard and Cruveilhier.

In the sixth chapter, the conditions demanding examination of the genital organs are discussed, and the necessity for such examination deduced from the following proposition, which formulates what we consider a radical principle of rational medicine:

"When the function of an organ is disturbed, the *prima facie* inference is that the organ itself, which constitutes the mechanism by which that function is performed, is out of gear." After the enunciation of a proposition so entirely in accord with the spirit of sound philosophy to be assumed by so distinguished an author as Dr. Barnes, we regret to find him qualifying it in the next sentence, as if startled at his own boldness: "This is not indeed always absolutely true, because an impaired state of the blood, or disordered innervation, or derangement of a different organ, may entail the functional disorder which arrests our attention." "The genital organs are no exceptions to this proposition." Nor, indeed, he should have added, are any other, and thus his first proposition would have comprehended the whole range of pathology.

Five chapters are devoted to the various discharges from the genital organs, in which category are included successively those consisting of mucus, water, air, pus and blood.

In the chapter on the "Significance of Pain," the author summarizes his definition of neuralgia and of hysteria in language deserving perpetuation, as "phases of aberrant, nervous action, the result of nervous exhaustion from disease, and mal-nutrition."

In the next chapter (XIII) the author coins a word which will doubtless some day be engrafted into medical technology, like its congener "dyspepsia," *i. e.*, "dyspareunia," difficult copulation.

His description of instruments, aptly headed "The Gynæcologist's Bag," is very complete, and the mode of their application given in detail and graphically illustrated.

The relations of the two phenomena of ovulation and menstruation are very clearly defined.

The determination of the duration of the menstrual function is withdrawn from the domain of popular tradition and dogma, and based upon physiological premises. Quoting Negrier, he says: "It seems well proved that the ovarian function, creative of germs, is prolonged in life in the direct ratio of the volume of the ovaries and of the precocity of ovulation."

The morbid conditions of the genital organs are treated successively in the order of their pathological importance, from those of the ovaries to those of the vulva. Time and space fail us for the further analysis of this work, a complete gynæcological library in itself, without which no gynæcological library would be complete.

H.

BOOKS RECEIVED.

*A Manual of Toxicology;* including the Consideration of the Nature, Properties, Effects, and Means of Detection of Poisons, more especially in their Medico-Legal Relations. By JNO. J. REESE, M.D., Professor of Medical Jurisprudence and Toxicology in the University of Pennsylvania. Philadelphia: J. B. Lippincott & Co. 1874.

*A Treatise on Therapeutics;* comprising Materia Medica and Toxicology, with especial reference to the Application of the Physiological Action of Drugs to Clinical Medicine. By H. C. WOOD, Jr., M.D., Professor of Botany, etc. Philadelphia: J. B. Lippincott & Co. 1874.

*Ligation of Arteries.* By Dr. L. H. Farabeuf, Aide d'Anatomie a la Faculte, ancien interne des Hopitaux de Paris. Translated by JNO. JACKSON, M.D., of Danville, Kentucky. Philadelphia: J. B. Lippincott & Co. 1874.

*The Science of Homœopathy;* or, a Critical and Synthetical Exposition of the Doctrines of the Homœopathic School. By CHARLES J. HEMPEL, M.D., etc., etc. Bœricke & Tafel, New York, etc. 1874.

*Manual of Eye Surgery.* By A. J. HOWE, A.M., M.D., Author of a Treatise on Fractures and Dislocations, etc., etc. Cincinnati: Wilstach, Baldwin & Co. 1874.

PAMPHLETS RECEIVED.

*The Annual Report of the Massachusetts Eye and Ear Infirmary.* 1874.

*The Anatomical, Pathological and Surgical Uses of Chlcral.* Read before the Pathological Society of Philadelphia, March 12, 1874, by W. W. KEEN, M.D., Lecturer on Anatomy in the Philadelphia School of Anatomy. Philadelphia: J. B. Lippincott & Co. 1874.

*The Theory and Art of Bread-Making, Without the Use of Ferment.* By Prof. E. N. HORSFORD.

*Transactions of the Fourth Annual Session of the Medical Society of Virginia.*

*Remarks on the Management of the Intermaxillary Bone in Double Hair Lip.* By W. R. WHITEHEAD, Denver, Colorado.

*Herpes Gestationis;* a Rare Affection of the Skin, Peculiar to Pregnancy. By L. DUNCAN BULKLEY, M.D. New York: William Wood & Co. 1874.

*Extract from a Report on the History of the Surgery of Tennessee,* comprising the subjects of Stone in the Bladder, Ovarian Tumors, Vesico-Vaginal Fistula. Made to the Tennessee State Medical Society, by Wm. T. BRIGGS, M.D., Professor of the Principles and Practice of Surgery, in the University of Nashville.

*Boylston Medical Society, of Harvard University.* Catalogue. March, 1874.

*Climate in Pulmonary Consumption, and California as a Health Resort.* By LEWIS ROGERS, M.D. Louisville: Jno. P. Morton & Co. 1874.

### JOURNALS RECEIVED.

The American Journal of the Medical Sciences—April.
" American Practitioner—April and May.
" Atlanta Medical and Surgical Journal—May.
" Boston Journal of Chemistry—May.
" Buffalo Medical and Surgical Journal—April.
" Chicago Journal of Nervous and Mental Disease—April.
" Clinic, Cincinnati—April 18, 25, May 2, 9.
" Cincinnati Lancet and Observer—May.
" Canada Medical and Surgical Journal—May.
" Druggists' Circular—April and May.
" Detroit Review of Medicine and Pharmacy—April and May.
" Dental Cosmos—May, 1874.
" Indiana Journal of Medicine—April and May.
" London Lancet—April, 1874.
" Medical Examiner, Chicago—April 15, May 1.
" Medical Times, Philadelphia—April 18, 25, May 2, 9.
" Medical and Surgical Reporter, Philadelphia—April 18, 25, May 2, 9.
" Medical News and Monthly, Philadelphia—April.
" Medical Herald (Leavenworth)—April and May.
" Medical Record, New York—April 15, May 1.
" Medical Press and Circular, London—April 15.
" New York Medical Journal—May.
" Northwestern Medical and Surgical Journal—May, 1874.
" Nashville Journal of Medicine and Surgery—April.
" Pharmacist—May, 1874.
" Practitioner, London—April, 1874.
" Le Progres Medicale, Paris—Nos. 11, 12, 13, 14, 15.
" Richmond and Louisville Medical and Surgical Journal—May, 1874.
" St. Louis Medical and Surgical Journal—May, 1874.
" Southern Medical Record—April, 1874.
" Virginia Medical Monthly, Richmond—Vol. 1, Nos. 1, 2, April and May.

